# Multiple Response Mechanisms of Plants to Drought Stress

**DOI:** 10.3390/plants13202918

**Published:** 2024-10-18

**Authors:** Jie Gao, Jie Zhao, Peijian Shi

**Affiliations:** 1 College of Life Sciences, Xinjiang Normal University, Urumqi 830054, China; 2 College of Resources and Environment, Linyi University, Linyi 276000, China; 3 Bamboo Research Institute, Nanjing Forestry University, Nanjing 210037, China

As climate change increasingly affects global ecosystems, understanding plant responses to drought stress has become essential for both conservation and agricultural productivity. Recent studies provide critical insights into how various plant species, including *Schoenus ferrugineus* and different Arabica coffee genotypes, adapt to water scarcity. Fedorov et al. employed MaxEnt modeling to predict the diminishing suitable habitats for *Schoenus ferrugineus* under climate change scenarios, highlighting the vulnerability of calcareous mires [1]. This study complements the findings of Chekol et al., who explored the physiological and metabolic responses of Arabica coffee, revealing significant correlations between drought tolerance and physiological traits across various genotypes [2]. Together, these studies underline the urgent need for conservation strategies that address the unique challenges faced by plants in rapidly changing climates.

Furthermore, research on biomass allocation in grasslands by Wang et al. emphasizes the influence of climate factors on the distribution of biomass between aboveground and belowground components. Their work shows that soil nutrient availability, shaped by climatic variations, significantly affects grassland productivity [3]. This understanding is vital for predicting future dynamics in these ecosystems, particularly in light of increasing drought events. In a related vein, Bao et al. investigated the role of the WRKY transcription factor BnWRKY49 in ramie, finding that its overexpression enhances drought resistance [4]. Similarly, Saimi et al. characterized the GhNAC2-A06 transcription factor in cotton, establishing its crucial role in drought response, which could lead to the development of more resilient cotton varieties [5].

The studies also delve into morphological and physiological adaptations that optimize water use efficiency. Sun et al. revealed an inverse relationship between stomatal density and average nearest neighbor distance in *Photinia* species, suggesting that the spatial arrangement of the stomata plays a critical role in optimizing gas exchange under drought conditions [6]. This relationship is crucial for enhancing carbon absorption while conserving water. Moreover, Jia et al. developed a model utilizing solar-induced chlorophyll fluorescence to assess photosynthetic responses in winter wheat experiencing drought stress [7]. Their findings highlight the potential for using fluorescence measurements as predictive tools for plant productivity in water-limited environments.

Lastly, Huang et al.’s research on leaf area distribution in drought-tolerant landscape plants offers insights into how competitive dynamics influence ecological design. By quantifying leaf area distribution inequality, their study provides valuable metrics for understanding how environmental pressures shape plant architecture [8]. Collectively, these studies illustrate the multifaceted nature of plant responses to drought stress, emphasizing the importance of genetic, physiological, and ecological factors in fostering resilience. As we navigate the challenges posed by climate change, these insights pave the way for innovative strategies in agriculture and conservation aimed at sustaining both plant and ecosystem health.
